# Analysis of Surface Protein Expression Reveals the Growth Pattern of the Gram-Negative Outer Membrane

**DOI:** 10.1371/journal.pcbi.1002680

**Published:** 2012-09-27

**Authors:** Tristan S. Ursell, Eliane H. Trepagnier, Kerwyn Casey Huang, Julie A. Theriot

**Affiliations:** 1 Department of Bioengineering, Stanford University, Stanford, California, United States of America; 2 Department of Biochemistry, Stanford University School of Medicine, Stanford, California, United States of America; 3 Department of Microbiology and Immunology, Stanford University School of Medicine, Stanford, California, United States of America; 4 Howard Hughes Medical Institute, Stanford University School of Medicine, Stanford, California, United States of America; University of Notre Dame, United States of America

## Abstract

The outer membrane (OM) of Gram-negative bacteria is a complex bilayer composed of proteins, phospholipids, lipoproteins, and lipopolysaccharides. Despite recent advances revealing the molecular pathways underlying protein and lipopolysaccharide incorporation into the OM, the spatial distribution and dynamic regulation of these processes remain poorly understood. Here, we used sequence-specific fluorescent labeling to map the incorporation patterns of an OM-porin protein, LamB, by labeling proteins only after epitope exposure on the cell surface. Newly synthesized LamB appeared in discrete puncta, rather than evenly distributed over the cell surface. Further growth of bacteria after labeling resulted in divergence of labeled LamB puncta, consistent with a spatial pattern of OM growth in which new, unlabeled material was also inserted in patches. At the poles, puncta remained relatively stationary through several rounds of division, a salient characteristic of the OM protein population as a whole. We propose a biophysical model of growth in which patches of new OM material are added in discrete bursts that evolve in time according to Stokes flow and are randomly distributed over the cell surface. Simulations based on this model demonstrate that our experimental observations are consistent with a bursty insertion pattern without spatial bias across the cylindrical cell surface, with approximately one burst of ∼10^−2^ µm^2^ of OM material per two minutes per µm^2^. Growth by insertion of discrete patches suggests that stochasticity plays a major role in patterning and material organization in the OM.

## Author Summary

All Gram-negative bacteria share common structural features, including an inner membrane, a stiff cell wall, and an outer membrane. Balancing growth in all three of these layers is critical for bacterial proliferation and survival, and malfunctions in growth often lead to cellular deformations and/or cell death. However, relatively little is known about how the incorporation of new material into the outer membrane is regulated in space and time. This work combines time-lapse microscopy with biophysical modeling and simulations to examine potential mechanisms by which new material is added to the outer membrane of the rod-shaped Gram-negative bacterium *Escherichia coli*. Our results indicate that the outer membrane grows in discrete bursts randomly distributed over the cylindrical cell surface. Each insertion event adds a random amount of new material, pushing old material into new locations and thus expanding the cell membrane. Using our biophysical model, we generated simulated fluorescence images and directly compared analyses of our experimental and computational results to constrain the rate and size of bursts of growth. Together, this indicates that growth of the outer membrane does not require spatial regulation, and the stochastic nature of insertion may contribute to the establishment of cellular patterning and asymmetry.

## Introduction

The Gram-negative outer membrane (OM) is a complex [1], non-uniform [2], largely immobile [3] collection of lipids, lipopolysaccharides (LPS), and membrane proteins. This asymmetric organelle is composed of phospholipids and lipoproteins in the inner leaflet, OM proteins spanning the membrane, and LPS in the outer leaflet. Proteins make up approximately two-thirds of the mass of the OM [4], and several OM proteins exhibit distinct subcellular localizations on the bacterial surface (polar, septal, or uniform) [5]. While there is a growing appreciation for the large diversity of outer membrane protein localization patterns [5], [6], *insertion* patterns have been elucidated only in a few special cases, such as the polar secretion of IcsA in *Shigella flexneri* [7], [8]; a general understanding of insertion patterning remains lacking. Although the spatiotemporal dynamics of OM protein insertion patterns have typically not been quantitatively measured, electron microscopy studies have shown that newly inserted porins in *Salmonella typhimurium* appear as discrete clusters [3], and also that LPS arrives in localized patches [9], [10], indicating that growth is the product of discrete events in which many molecules are inserted in bursts.

In Gram-negative bacteria, lipids, proteins, and LPS must traverse the inner membrane and the periplasmic space before insertion into the OM, and each step could potentially be spatially localized and/or occur in bursts. Many components of the molecular machinery implicated in OM protein and LPS transport have only recently been identified [11]–[20]. Secreted proteins are synthesized in the cytoplasm and tagged with an N-terminal signal peptide that targets them for transport across the inner membrane via either the Sec or Tat pathways [21], both of which are widely conserved among bacteria. The Sec apparatus is uniformly distributed in the inner membrane, while the Tat pathway is concentrated at the poles; nevertheless, some polar-targeted proteins such as IcsA [22] are transported through the Sec apparatus. Finally, translocation across the cell wall and insertion of folded proteins into the outer membrane is mediated by the BAM (β-barrel assembly machinery) complex [21], [23].

After delivery, the dynamic behavior of OM proteins varies according to subcellular position. Label-and-chase experiments, in which cells are imaged immediately after fluorescent labeling of OM proteins and again during growth without further labeling, show cells that initially have uniformly bright peripheries (indicating that OM proteins are distributed reasonably uniformly across the surface at high density) but transition over several generations to non-uniform fluorescence distributions, ultimately with only originally labeled “old" poles (poles of progenitor bacteria, versus new poles synthesized during subsequent rounds of bacterial division) remaining bright [24]. While general labeling of all outer membrane proteins using amine-reactive (succinimidyl ester-linked) fluorescent dyes revealed that a subset was freely diffusible, the non-uniform pattern after label-and-chase indicated that other proteins were far less mobile [25]. Similarly, lectin-labeled LPS molecules were virtually immobile on the time scale of growth [25], although crosslinking by the multivalent lectin might have limited LPS mobility in this experiment. Since time intervals on the order of one cell cycle are required to produce a shift in cellular fluorescence distribution, growth itself may be intimately coupled to the localization of older OM proteins. The simplest interpretation of polar retention is that new OM material is inserted along the cylindrical portion of the cell but not at the poles. Thus, material at poles tends to remain at the poles, while older material in the cylindrical portion of the cell is spread out by the insertion of new material that results in growth.

To elucidate the role of growth in OM organization, we examined the spatial pattern of initial secretion and subsequent redistribution of the abundant OM protein LamB (maltoporin) in live *Escherichia coli* cells. LamB is responsible for the uptake of maltose or maltodextrins, which are important carbon sources and the primary breakdown products of starches in the human intestine [26]. This channel protein also transports other carbohydrates including glucose, lactose, and glycerol [27], [28], and is the receptor for bacteriophage λ [29], [30]. In this work, we study the underlying growth pattern of the *E. coli* OM on generational time scales by adding a single, small, covalently bound fluorophore to LamB. We found that LamB is secreted in discrete punctate spots that diverge from one another during cell elongation, but are virtually immobile in the absence of cellular growth. LamB puncta that are positioned at the poles during septal growth and subsequent division are retained at the poles, consistent with the polar retention characteristic of the OM population as a whole. We used computational modeling of OM growth to demonstrate that the motion of these puncta, including the dilution of older OM components in the cylindrical portion of the cell, may result from discrete insertion events that are temporally stochastic and randomly distributed over the cylindrical cell surface.

## Results

### Covalent single-fluorophore labeling of surface-exposed LamB

Our experiments employed a genetically encoded 20-residue peptide tag inserted into a surface-exposed external loop of the OM protein LamB [31]. This peptide is recognized and covalently labeled with a single tetramethylrhodamine-Coenzyme A (TMR-CoA) fluorophore by the enzyme Sfp [31]. Sfp labeling has previously been demonstrated to label proteins in solution and on mammalian cell membranes [31]–[33]; this is the first application of this technique to an *E. coli* surface protein. Fluorescent labeling of the bacterial surface expressing LamB under an IPTG-inducible promoter from a multi-copy plasmid required the presence of the peptide tag, Sfp enzyme, and TMR-CoA. SDS-PAGE analysis revealed covalent attachment of the fluorophore to only a single protein band, corresponding to the size of LamB (data not shown).

The importance of the biological processes associated with LamB has motivated a diverse collection of genetic and biochemical investigations of the function and behavior of LamB at the level of single proteins, including tracking and mobility of the LamB distributions in live cells [34]–[36]. The Sfp labeling technique is well suited to study the motion of bacterial OM proteins, for which the most common protein visualization technique, Fluorescent Protein (FP) fusions, fails because FPs do not fold properly when exported through the general secretory apparatus [37]. Like FP fusions, our labeling strategy uses a covalently attached molecule suitable for following proteins over generational timescales. This probe has the additional advantage that the peptide tag and covalently attached fluorophore are physically small compared to FPs and to other probes previously used to study LamB surface motion [34]–[36], and is therefore minimally perturbing and offers higher spatial resolution.

### Newly secreted LamB proteins form stable fluorescent puncta in the OM

**Figure 1:**
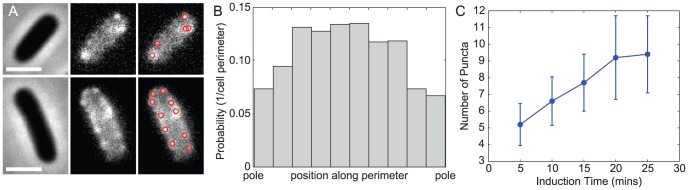
LamB puncta initially appear at a constant rate. (A) Left to right, phase contrast, fluorescence, and algorithm-detected spots for two typical bacteria at 5 minutes (top) and 25 minutes (bottom) after induction. Scale bars are 2 um. (B) Distribution of puncta locations after 15 minutes of induction as a function of normalized cell length for 165 cells. (C) Mean number of puncta per cell as a function of induction time, averaged over 440, 158, 165, 227, and 228 cells at the 5-, 10-, 15-, 20-, and 25-minute time points, respectively. Error bars indicate one standard deviation above and below the mean.

After IPTG induction of the modified LamB for at least five minutes and subsequent covalent labeling, we observed punctate fluorescence distributions of new protein across the cell surface (Fig. 1A,B). The number of fluorescent puncta per bacterium initially increased linearly as a function of induction time (Fig. 1C); however, for induction times greater than 20 minutes, the number of distinguishable puncta began to plateau (Fig. 1C) due to overlap in the fluorescence distributions of newly appearing puncta with those already present on the bacterial surface.

**Figure 2:**
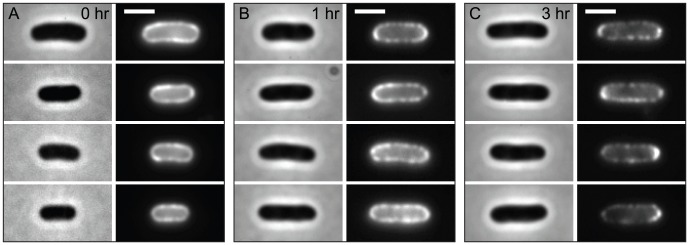
In bulk label-and-chase experiments, LamB puncta eventually localize at the poles. In each frame, phase-contrast images are on the left, fluorescence images are on the right. Scale bars are 2 µm. Cells were induced for 1 hour prior to labeling. (A) Immediately after labeling, the LamB fluorescence is uniform around the cell periphery rather than punctate. (B) Distinct puncta have appeared after an hour of growth post-labeling. (C) After 3 hours of growth, puncta along the cell periphery were well separated, while a fraction of the labeled LamB clearly showed polar localization, due to lack of growth in the polar region.

After 1 hour of induction, the fluorescent label distribution on the cell peripheries appeared to be nearly uniform (Fig. 2A). When these cells were allowed to grow in the absence of inducer, dark zones of unlabeled material appeared and were accompanied by the emergence of fluorescent puncta from groups of older, labeled protein. Unlike puncta created by brief induction and subsequent labeling, these puncta were produced by the dilution of labeled material into optically resolvable regions by insertion of new, unlabeled material (Fig. 2B,C). This unlabeled material, which includes LamB, other OM proteins, and LPS, must be inserted in discrete bursts to produce the observed puncta.

**Figure 3:**
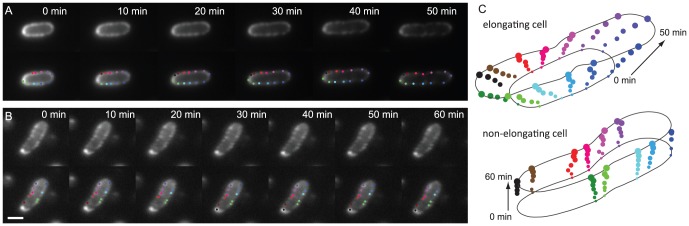
Puncta motion is only observed in elongating cells. (A,B) Cells and identified puncta (colored dots) as a function of time for elongating (A) and rifampin-treated, non-elongating cells (B). Scale bars are 2 µm. (C) Positions of puncta for the cell in (A) diverge as the cell elongates over time, while the positions of the puncta for the non-elongating cell in (B) are relatively static.

These observations suggested that OM growth drives the movement of labeled LamB puncta. To examine this hypothesis directly, we used the RNA polymerase inhibitor rifampin to block new mRNA synthesis, thereby arresting cell growth. Bacteria grown in the presence of inducer, fluorescently labeled, and then treated with rifampin exhibited stationary fluorescence distributions for 60 minutes, with no apparent broadening or large-scale migration of the puncta (Fig. 3B). Consistent with previous experiments [34]–[36], [38], LamB puncta were essentially immobile in non-elongating, rifampin-treated cells (Fig. 3B,C). However, when driven by the dynamics of cell growth, puncta moved apart (Fig. 3A,C). In addition, fluorescence recovery after photobleaching of rifampin-treated cells revealed that fluorescent puncta do not recover by 40 minutes after photobleaching (data not shown). These observations suggest that puncta are structures that do not engage in extensive diffusive motion or molecular exchange, and thus indicate that there is no large-scale motion of LamB molecules on the surface of non-elongating *E. coli*.

### LamB puncta exhibit growth-dependent motion

In contrast to rifampin-treated cells, the fluorescence distributions on the surfaces of growing cells changed substantially over time (Fig. 3A,C). Initially, bacteria induced to express tagged LamB for 1 hour exhibited a uniform peripheral fluorescence distribution after labeling (Fig. 2A). As the cells elongated, two notable shifts in the fluorescence distribution occurred. First, distinct fluorescent puncta emerged along the cylindrical portion of the cell periphery. Second, the cylindrical walls of the bacteria became progressively dimmer over successive rounds of division. In contrast, labeled OM material that either started in the polar regions, or that became incorporated into the polar regions during the division process, formed persistently bright puncta at the poles, indicative of the “polar retention" that is characteristic of the OM population as a whole (Fig. 2C,3A) [2].

To measure the growth-dependent dynamics of fluorescent LamB puncta in single cells, we tracked puncta with time-lapse epifluorescence microscopy and measured the divergence of adjacent puncta around the cell periphery. Puncta along the cylindrical walls of the cell diverged from their neighbors faster than puncta in the polar regions (Fig. 3A,C). Movements of puncta tended to be directed, as expected for the surface expansion of an elongating cell. In addition to pairwise divergence, many fluorescent puncta exhibited broadening, and occasionally a single punctum split into two distinguishable spots. We hypothesized that these effects were due to different degrees of insertion of dark material (unlabeled LamB and other material) into a diffraction-limited punctum of fluorescent material (labeled LamB). In the case of broadening, if a small or finely distributed amount of dark material is inserted into a diffraction-limited region, the fluorescence distribution may broaden, but not to the point of becoming two individually resolvable puncta. In contrast, spot splitting may occur when enough dark material is inserted into a diffraction-limited fluorescence distribution to split the fluorescence into two distinguishable puncta. Given the negligible mobility of puncta in rifampin-treated cells (Fig. 3B,C), our observations suggest that divergence of neighboring puncta, puncta broadening, and occasional puncta splitting are all due to the pattern of incorporation of new OM material, rather than due to diffusion. Likewise, our data suggests that material incorporated near midcell during the division process remains there over generational time-scales once this region becomes a new pole, due to the significantly reduced rate of insertion at the poles.

### Computational modeling of OM insertion recapitulates puncta behavior

Based on our observations that the motion of LamB puncta is dependent on growth, we hypothesized that the patchy incorporation of LamB in the OM could be representative of the pattern of OM incorporation as a whole. Our own measurements of LamB mobility as well as measurements by others [3] suggest that diffusion is not a major protein transport mechanism within the OM. In addition, on the time scales of interest for cell growth, we assume bilayer viscoelasticity is negligible. With these constraints in mind, we constructed a minimal model of the OM as a viscous, incompressible, two-dimensional fluid without diffusion that moves with laminar flow. In this model, the only motion relevant on the time scale of cell doubling is due to insertion. Insertion of new material causes elongation of the cell body, which we assume remains cylindrical with a fixed radius matching the shape of the cell wall. Our model of growth does not explicitly include the hemispherical poles, however, the cylindrical shape constraint dictates that there is necessarily a stable, convergent zone of material flow at each end of the cell, out of which material cannot flow in the absence of active insertion in that region.

Simulations based on our model consist of a temporally stochastic sequence of insertion events occurring randomly across the cylindrical cell surface. The overall process of insertion is controlled by three kinetic parameters, *k_on_*, *k_ins_*, and *τ*. We assume that each insertion event is comprised of three steps. First, insertion events are initiated at a fixed mean rate per unit area (*k_on_*) following Poisson statistics. Each insertion event occurs independently and is uniformly distributed over the cylindrical cell surface. Second, insertion at a particular site leads to the addition of new material at a fixed rate (area per unit time) of *k_ins_*. Third, each insertion event proceeds for an exponentially distributed duration *τ* corresponding to a single kinetic step for terminating insertion. Note that these assumptions are not critical for the conclusions of the model, rather they were selected as the simplest possible set of assumptions. Each insertion event creates a patch of material with average area *τ k_ins_* that is labeled ‘light’ during the simulated induction phase, or ‘dark’ during insertion after the induction phase (see Materials and Methods). We note that our model can easily be modified to account for a non-uniform spatial distribution of insertion locations or a non-exponential distribution of insertion-event initiation and duration times; however, this model allows us to explore a wide range of spatial patterns that can be compared with our experimental data.

The average behavior of this stochastic growth process is described by the coupled first-order differential equations
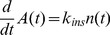
(1)

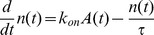
(2)where *A*(*t*) is the area of the cylindrical cell and *n*(*t*) is the number of insertion sites at time *t*. In Eq. 1, the OM area increases at a rate proportional to the current number of insertion sites and the insertion rate. In Eq. 2, the number of insertion sites increases proportional to the current cell area and the insertion initiation rate, equivalent to the assumption that the average concentration of insertion sites is constant, with insertion terminating in a single kinetic step. The solutions to Eq. 1 and 2 describe growth according to the exponential function 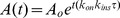 with a doubling time 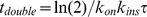, where *A_o_* is the initial area of the cylindrical portion of the OM.

To simulate the full flow field of the OM as the cell grows, our model of OM growth discussed above can be couched as a material flux conservation problem. Given the locations of point sources of new material insertion, 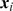, the material flux field follows the continuity equation
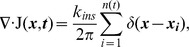
where 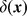 is the Dirac delta function, and 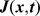 is the vectorial material flux over the cell surface in time. Each insertion site creates a linearly-independent flow field given by
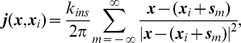
where the image vectors 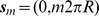 map the 2D plane onto the cellular topology of a wrapped cylinder with radius *R*. The position of each insertion site, 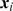, is then updated by the total flux field, given by
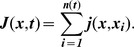
Using this stochastic model, we performed simulations to mimic the conditions of our label-and-chase experiments in which light material was inserted for 15 minutes, followed by 75 minutes of dark-material insertion. To determine the kinetic parameters for which our simulations most closely match our experimental observations, we explored the growth dynamics in a large region of parameter space across more than four orders of magnitude in *k_ins_* and three orders of magnitude in *k_on_*. To match our experimental measurements, we fixed the doubling time at *t_double_* = 90 minutes, which constrains the average duration for an insertion event to 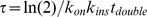. A lower bound on the rate of initiation of insertion events was determined from our induction data (*k_on_*>0.0005 µm^−2^ s^−1^, Fig. 2C), and we estimated the lower bound on the rate of material insertion to be *k_ins_* = 10 nm^2^ s^−1^. Using our model, we scanned values of the average insertion event duration from 0.1 s<*τ*<200 s and average area per insertion event from 200 nm^2^<*τ k_ins_*<1 µm^2^. Thus, the range of mean insertion sizes spanned from well below to well above the diffraction limit.

**Figure 4:**
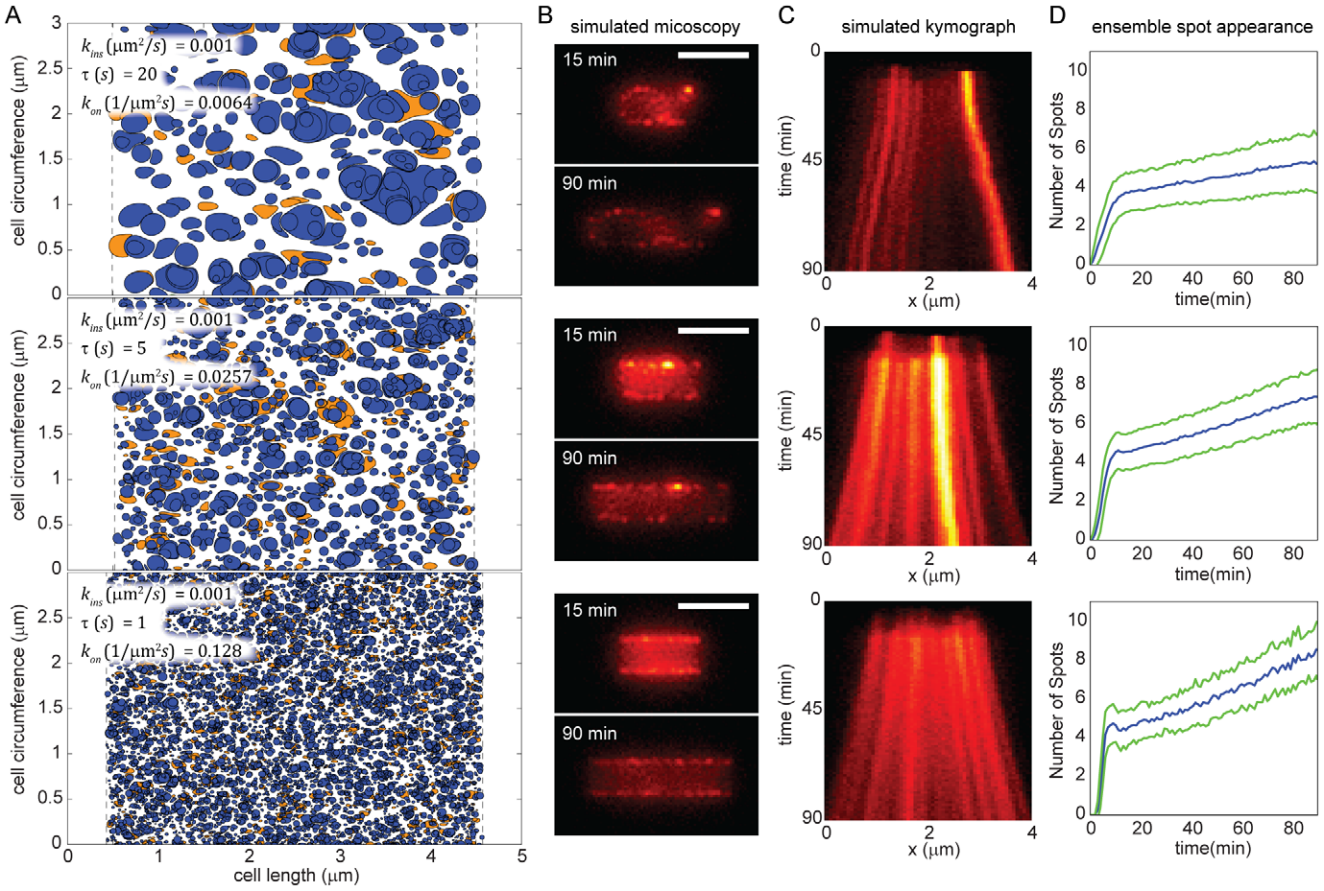
The mean area of insertion events is a determinant of the spatial distribution of material in the OM. (A) Three simulations of the OM after 90 minutes of growth, with the same doubling time but differing kinetic parameters as indicated. Orange regions indicate labeled (bright) material inserted for the first 15 minutes. Blue regions indicate unlabeled (dark) material inserted after the first 15 minutes. Interstitial white areas are the original OM material at *t* = 0. The boundaries of new growth (vertical dashed lines) are where the flow field symmetrically converges to an inert polar region not included in our simulations. (B) Simulated fluorescence microscopy of configurations in (A) immediately after labeling and after 90 minutes of growth. Scale bar is 2 µm. (C) One-dimensional kymographs for the time-series data from the top edge of the simulated cells in (B), initially showing the insertion of labeled material, followed by growth and spreading of labeled puncta. (D) The number of puncta detected on the top cell edge as a function of time for the kinetic parameters in (A). The green lines are one standard deviation above and below the mean number of puncta, shown in blue. Each curve represents the ensemble average of at least 250 simulations using the same kinetics parameters as in (A).

The average area added in each insertion event, *τ k_ins_*, determined the degree of patchiness of the simulated fluorescent material in the OM. The effect of varying the mean insertion size over a modest range (20-fold variation) is shown in Fig. 4; note that changes in *τ* can be complemented by changes in *k_ins_* (and vice versa), so that only their product is constrained. In Fig. 4A, newly inserted light and dark material for large, medium, and small mean insertion areas are depicted by the polygons representing each insertion event, with the interstitial white space occupied by the original material. The clear vertical boundaries on the left and right sides of Fig. 4A, where no new material was inserted, are the zones where flow created by growth converges symmetrically on the polar zones, hence the relatively clear delineation between those inert polar regions and the cylindrical regions of active growth. In order to determine whether our model was consistent with our experimental data, we developed software [39] to simulate the full 3D fluorescence light field from our modeling results, along with the corresponding 2D micrographs (Materials and Methods). The configurations in Fig. 4A were convolved into simulated fluorescence images (15 min and 90 min time points shown in Fig. 4B), and a kymograph was generated using the fluorescence pattern along the top surface of the simulated cell (Fig. 4C). If the average area added during an insertion event was large, the motion of OM material was highly stochastic, and the length scale of OM heterogeneity between newer and older material was relatively large (Fig. 4, top panels). Conversely, if the average area added per insertion event was small, many insertion events were required to achieve the same degree of elongation; these insertion events were uniformly distributed over the cell surface and hence led to relatively even spreading of the OM, and a relatively short length scale of OM heterogeneity (Fig. 4, bottom panels). Thus, by varying the average area of each insertion event, a wide range of simulated fluorescence patchiness was accessible in our model.

### The rate of punctum appearance constrains model parameters

**Figure 5:**
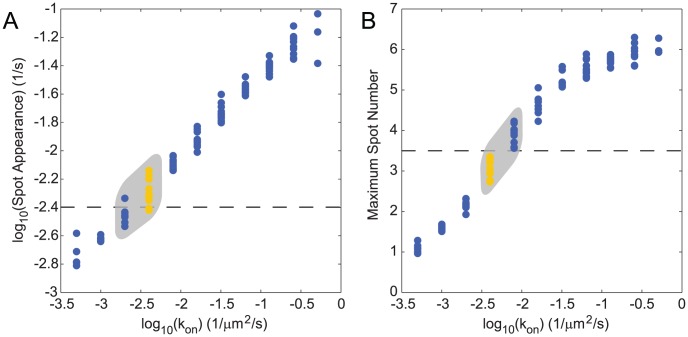
Simulated fluorescence and experimental microscopy data constrain the relevant region of parameter space. Average values of the initial punctum appearance rate (A) and maximum number of puncta normalized for cell perimeter length (B), as a function of *k_on_* for 101 parameter sets. The black dashed lines indicate values determined from experiment. The gray boxes indicate simulated data that is consistent with either the spot appearance rate or maximum spot number estimated from experimental data. The yellow data highlight parameter sets with values of *k_on_* that are consistent with experimental measures of both spot appearance rate and maximum number.

To further constrain the values of the activation rate *k_on_* and the mean insertion size *τ k_ins_*, we used our model to compare simulations and experiments using quantitative metrics motivated by our experimental punctum appearance data. We performed at least 21 independent simulations for 101 parameter sets to explore a wide range of values of *k_on_* and *k_ins_* (with *τ* constrained by the fixed doubling time), and calculated the average number of diffraction-limited puncta as a function of time, similar to Figs. 1C and 4D. We then fit a sigmoidal function to these curves to define the initial punctum appearance rate and maximum number of puncta (Fig. 5). When normalized for cell perimeter length and compared with the experimental data (Fig. 1C), both of these metrics predicted an activation rate of 0.002 µm^−2^ s^−1^<*k_on_*<0.008 µm^−2^ s^−1^ and a mean insertion size of 0.015 µm^2^<*τ k_ins_*<0.06 µm^2^, equivalent to the area of ∼1000 typical OM proteins. The proximity of this mean insertion size to the spatial resolution of the optical system, limited by the PSF, is consistent with the observation that puncta both broaden and split during experiments.

**Figure 6:**
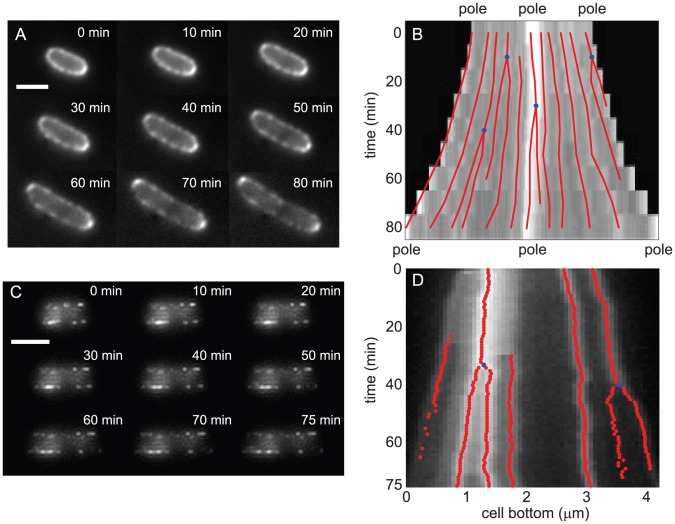
Viscous fluid model of OM dynamics recapitulates key experimental features of puncta appearance and motion. (A) Fluorescence images of an elongating bacterium induced for 1 hour and fluorescently labeled. Scale bar is 2 µm. (B) Kymograph of the cell periphery in (A) over approximately one cell cycle showing spots linked into tracks (red lines). The blue dots indicate spot splitting events. (C) Simulated fluorescence microscopy data of OM growth following our Stokes fluid model, with labeled LamB insertion for 15 minutes followed by unlabeled growth for 75 minutes. (D) Kymograph of the time series represented in (C). The qualitative features of punctum divergence and splitting from (B) are recapitulated in our computational model. Red lines indicate the positions of puncta determined with a spot-finding algorithm, and blue dots indicate splitting events.

To determine whether our computational model successfully reproduced other qualitative features of the experimental data, we focused on parameters that matched our experimental punctum appearance data and performed identical analyses on both real and modeled data to study puncta spatial distributions and motion. For *k_on_* = 0.004 µm^−2^ s^−1^, *k_ins_* = 0.00064 µm^2^ s^−1^, and *τ* = 50 s, our model produced punctate fluorescence distributions with growth causing divergence of puncta around the cell periphery (Fig. 6C) similar to experimental measurements (Fig. 6A). Our simulations also revealed that puncta splitting and broadening can be explained by the blurring effects of the point-spread function (PSF) on the fluorescence of labeled material after the insertion of dark (unlabeled) material (Fig. 6D). If a small amount of dark material whose size is below the diffraction limit was inserted within a bright region of the OM, the punctum subsequently appeared broader and dimmer, since the resulting pattern could not be resolved as two separate fluorescence distributions. However, if enough unlabeled material was inserted to create a dark region of size similar to the PSF width, the single fluorescent punctum split into two distinguishable puncta. Hence, the relative occurrence of punctum broadening and splitting was determined by the distribution of areas of insertion events. Taken together, our model suggests that patchy OM growth in the cylindrical portion of the cell can lead to the observed phenomena of punctate fluorescence, divergence of puncta, puncta broadening, puncta splitting, and the polar retention of OM proteins in general [24].

## Discussion

Our observations and simulations support a model in which OM growth occurs in discrete insertion events that are uniformly distributed over the cylindrical cell surface, forming an inhomogeneous mixture of newer and older OM on a length scale set by the size of the insertion events. The appearance of fluorescent puncta can be attributed to a large average area per insertion event (Fig. 4A), and simulations based on our fluid dynamics model indicate that the observed growth patterns do not require spatial organization of insertion events, mechanical stresses, or biochemical interactions between molecules. Similarly, the pattern of newly inserted porins in *S. typhimurium* observed using electron microscopy consists of patches with dimensions of 50–100 nm that increase in number over time but do not increase in size [3]. We observed this patchy insertion directly in our induction time-course experiments, in which longer induction times produced more puncta that were more densely packed on the cylindrical cell surface (Fig. 1), and indirectly in our growth experiments, in which new unlabeled material dispersed the older, labeled OM into punctate spots (Fig. 2). Additionally, other direct and indirect measurements indicate that active translocation events occur at discrete sites on the cylindrical cell surface [40]. While the cylindrical section of the cell undergoes patchy growth, at the division septum, OM material that was once part of the actively growing cylindrical portion of the cell becomes trapped in the inert polar region. Our experiments showed that at short induction times, where new insertion events resulted in new fluorescent puncta, LamB puncta appeared more often in the cylindrical portion of the cell than at the poles (Fig. 1B), indicating that the rate of insertion was indeed lower at the poles. This observation is consistent with our bulk label-and-chase experiments that showed faster dilution of the fluorescence distribution in the cylindrical region, and, after a few generations, the emergence of relatively bright “old" poles that resulted from the lack of growth in the polar region after the initial labeling (Figs. 2C and 3). While our data cannot be used to rule out the possibility that groups of molecules much smaller than the diffraction limit are occasionally being inserted, when combined with the mechanistic insights from our model, our data suggests that the formation, evolution, and movement of fluorescent OM puncta is well explained by fairly large (∼10^−2^ μm^2^) discrete insertion events that are uniformly distributed over the cylindrical cell surface, with an exponentially distributed duration that has a single, fixed time constant.

Within the production and transport pathway of proteins bound for the OM, several possible molecular mechanisms could produce the punctate growth pattern observed here. First, nearly all OM proteins (with the known exception of PulD [41], [42]) are transported from the periplasm, through the rigid cell wall, and inserted into the OM by BAM complexes, and hence one possible interpretation of our data is that the appearance of fluorescent LamB puncta reflects the location of active BAM complexes and OM protein insertion in general. A second, independent possibility is that the uniformly distributed Sec apparatuses [22], responsible for translocating proteins from the cytoplasm to the periplasm where BAM complexes then effect OM insertion, are only active at specific locations, and hence determine the pattern of protein insertion in the OM. A third interpretation arises from the recent discovery that chromosomally expressed mRNAs remain localized to their transcription sites and form diffraction-limited spots, with mRNA diffusion coefficients orders of magnitude lower than expected for free diffusion [43]. While this observation has not been replicated for plasmid-expressed mRNAs, several multicopy plasmids have been shown to localize to a handful of foci in *E. coli* [44]. The mean duration of an OM insertion event (2 to 30 sec) is considerably shorter than the half-life of *E. coli* mRNA (∼400 sec) [45], thus bursts of cell growth are likely not due to transient mRNA production. However, we speculate that the combination of plasmid localization and mRNA localization to a few foci per cell could give rise to localization of secreted LamB. This list is by no means exhaustive; as data on the lifetimes of candidate molecular complexes (e.g. BAM and Sec) become available, the regions of parameter space we have identified through screening of computational results (Fig. 5) could provide a useful means of distinguishing among possible mechanisms producing the patchy patterns of OM protein insertion.

Our observations suggest that bacterial cell surface organization is strongly influenced by growth patterning. Calculations based on ferritin labeling of newly translocated LPS in *Salmonella* predict that an LPS molecule would diffuse less than 300 nm [9], [10], or approximately one twelfth of the cylindrical circumference, on the time scale of bacterial cell division. These ferritin-labeled LPS molecules initially appear in clusters assumed to be membrane translocation sites. Electron micrographs taken at regular intervals after labeling revealed that these clusters gradually disperse from each other across the surface of *Salmonella*, beyond the 300 nm diffusion limit. In comparison to the doubling of OM area that occurs on this time scale, it is reasonable to expect that the motion of OM proteins is dominated by patterns of OM incorporation, not diffusion.

The spatial pattern of protein expression dictates not only the distribution of proteins within a single cell, but also the distribution of OM proteins in a population. The unequal partitioning of proteins into daughter cells can produce heterogeneity among isogenic bacterial populations, and in a growing bacterial population, the fraction of cells harboring ‘old poles’ shrinks exponentially. If an OM protein is expressed and subsequently repressed early in the growth curve, polar retention will cause it to be concentrated at these few old poles. In this manner, OM protein inheritance could generate heterogeneity based on generational age that has important phenotypic consequences for differential permeability to essential nutrients, drug concentration, or phage infection. The agreement between our simple model and the observed motion of LamB puncta suggests that heterogeneity may be a general outcome in any bacterium in which the OM grows in discrete bursts.

## Materials and Methods

### Bacterial strains

The background *E. coli* strain used in this study was a *lamB*::kan single-gene deletion strain from the Keio collection [46]. Strain JAT567 is this deletion strain transformed with plasmid pEHT1 encoding a LamB derivative under the lac promoter. pEHT1 was constructed from pSB2267 [47]. The 14-amino acid ybbR tag with flanking glycine residues (GGGTVLDSLEFIASKLAGGG) [31] is located between codons 155 and 156 of the mature LamB sequence, which were mutated to introduce *PstI* and *XhoI* sites, respectively [47]. Plasmid pSB2267 was linearized by digestion with *PstI* and *XhoI* and ligated with olignonucleotides containing the ybbR-tag sequence (ggt)_3_ accgttcttgattctcttgaatttattgctagtaagcttgcg(ggt)_3_.

### Expression and purification of Sfp phosphopantetheinyl transferase

Purified Sfp protein was a generous gift from Chun Tsai. Sfp was expressed from plasmid pRSG56 [48]. Cells were induced with 0.4 mM IPTG in 2YT for 18 hours at room temperature and lysed in an EmulsiFlex-C5 (Avestin Inc.). The cell lysate was centrifuged and the protein was purified from the filtered supernatant in two steps. Filtered lysate was purified by anion exchange with a HiTrap Q FF column (GE Healthcare) in 50 mM Bis-Tris (pH 6.5) with 2 mM EDTA and a linear gradient from 0 to 500 mM NaCl. Fractions were analyzed by SDS-PAGE and Coomassie staining. Fractions containing Sfp were pooled, concentrated, and purified by gel filtration over a Superdex 200 column (GE Healthcare) in 50 mM Tris-Cl [pH 8.0], 5% glycerol, and 10 mM MgCl_2_.

### Synthesis and purification of TMR-CoA

TMR-CoA was synthesized according to the protocol of Yin *et al.* [49] with the substitution of methanol buffers for HPLC purification. Purified TMR-CoA was a generous gift from Kierstin Schmidt.

### Labeling of LamB proteins on bacterial cell surfaces

LamB was labeled using Sfp, which covalently attaches a TMR-CoA conjugate to the ybbR tag [31]. Mid-logarithmic phase JAT567 cells were labeled in a 50-µL volume with 1.5 µM Sfp, 5 µL of 12 µM TMR-CoA, and 10 mM MgCl_2_ at room temperature for 30 minutes. After labeling, the cells were washed and resuspended in Luria-Bertani broth (LB) at room temperature.

### Live-cell microscopy of fluorescently labeled LamB

Strain JAT567 was diluted 1∶100, grown to mid-logarithmic phase in LB with 35 µg/mL chloramphenicol for 1.5 hours at 37°C, and induced with 1 mM IPTG. Cells were then washed in LB and labeled with TMR-CoA. For measurements of the effects of LamB induction time, cells were imaged immediately on a Zeiss Axioplan 2 (Zeiss, Thornwood, NY) fluorescence microscope, using a MicroMAX 512BFT (Princeton Instruments, Trenton, NJ) camera with 6.8 µm pixels and a 100X 1.4NA objective, and captured with the MetaMorph (Molecular Devices) software package. For label-and-chase experiments, cells were grown at room temperature for 1 hour before imaging. One microliter of labeled cells was mounted on an agar pad and sealed with 1∶1∶1 vaseline∶lanolin∶paraffin, and the cells were imaged under phase contrast and epifluorescence at room temperature as described in [36]. For observations of protein movement in the absence of cell growth, cells were induced and labeled as described above and then incubated in LB with 10 µg/mL rifampin at room temperature for ten minutes and imaged on rifampin agarose pads.

### Tracking labeled LamB

We used the Matlab (The Mathworks, Inc., Natick, MA) package PSICIC to define cell outlines and intracellular coordinate systems based on phase images [50]. Corresponding fluorescence images of each bacterium were input into the MTT-peak detection algorithm [51] to determine the positions of fluorescent puncta. Puncta further than four pixels (∼272 nm) from the cell border were removed from further analysis. The remaining fluorescence peaks were linked into multi-frame tracks in two dimensions using the u-track software [52]. These tracks were ordered sequentially around the perimeter and projected onto the cell border for display in one-dimensional kymographs using ImageJ.

### Simulations of OM growth

Using a custom algorithm written in MatLab, we modeled the OM as a cylinder whose surface is a two-dimensional viscous Stokes fluid without diffusion. Each new insertion event began at a random location on the cylinder at a fixed rate per unit area. Each insertion event had an exponentially distributed duration, during which new material was inserted at a fixed rate of area per unit time. Newly inserted material was labeled as either ‘light’ or ‘dark’ to mimic labeling conditions in our experiments. Each new insertion event resulted in the creation of a small, regular polygon whose size and number of vertices were determined by the kinetic rates of area insertion and event duration. To model the effect of Stokes flow on the newly inserted material, the positions of the polygons' vertices were evolved in time using a time-dependent Green's function that was calculated using a series expansion of source images truncated symmetrically at 21 total terms. The simulation time step was chosen to ensure proper sampling of the exponential distribution of insertion-event duration times.

For each configuration of polygons, simulated fluorescence images were generated by convolution with the three-dimensional point spread function for the appropriate optical properties of our experimental data acquisition (100X 1.4NA microscope objective at 575 nm on a charge-coupled device with simulated noise and a pixel size of 6.8 µm).
